# Etiology of Thrombocytosis in a General Medicine Population: Analysis of 801 Cases With Emphasis on Infectious Causes

**DOI:** 10.4021/jocmr1125w

**Published:** 2012-11-11

**Authors:** Stacey R. Rose, Nancy J. Petersen, Tracie J. Gardner, Richard J. Hamill, Barbara W. Trautner

**Affiliations:** a National Institutes of Health, National Institute of Allergy and Infectious Diseases, 10 Center Drive, Bethesda, MD 20892, USA; b VA HSR&D Houston Center of Excellence, Michael E. DeBakey VA Medical Center (MEDVAMC 152), 2002 Holcombe Blvd., Houston, TX 77030, USA; c Department of Medicine, Health Services Research Section, Baylor College of Medicine, Houston, TX 77030, USA; d Menninger Department of Psychiatry and Behavioral Sciences, Baylor College of Medicine, One Baylor Plaza, Houston, TX 77030, USA; e Michael E. DeBakey VA Medical Center, Houston, TX 77030, USA; f Houston VA HSR&D Center of Excellence, Michael E. DeBakey VA Medical Center, 2002 Holcombe Blvd., Houston, TX 77030, USA; g Department of Medicine, Infectious Diseases Section, Baylor College of Medicine, One Baylor Plaza, Houston, TX 77030, USA

**Keywords:** Thrombocytosis, Elevated platelet count, Secondary thrombocytosis, Infection

## Abstract

**Background:**

The clinical importance of an elevated platelet count is often overlooked, particularly as a diagnostic clue to the presence of an underlying infection. We sought to better describe the relationship between thrombocytosis and inflammatory conditions, with a focus on infectious causes.

**Methods:**

We retrospectively reviewed 801 sequential cases of thrombocytosis (platelet count > 500 × 10^9^/L) at a tertiary care hospital.

**Results:**

Essential thrombocythemia was the most common cause of primary thrombocytosis, and these patients were more likely to have extreme (> 800 × 10^9^/L) and prolonged (> 1 month) thrombocytosis. Secondary thrombocytosis was more common than primary, with infectious causes accounting for nearly half the cases. Demographic factors associated with an infectious etiology included inpatient status, quadriplegia/paraplegia, an indwelling prosthesis, dementia and diabetes. Clinical and laboratory characteristics associated with an infectious cause of thrombocytosis included fever, tachycardia, weight loss, hypoalbuminemia, neutrophilia, leukocytosis and anemia. Patients with thrombocytosis secondary to infection had a more rapid normalization of platelet count, but higher risk of dying, than those with secondary, non-infectious causes.

**Conclusions:**

Infection is a common cause of thrombocytosis and should be considered in patients with comorbidities that increase risk of infection and when clinical and/or laboratory data support an infectious etiology. Thrombocytosis may have prognostic implications as a clinical parameter.

## Introduction

The laboratory finding of thrombocytosis, defined as an abnormally elevated platelet count, is not generally recognized as a clinical indicator of infection. However, our clinical experience has suggested that patients with an undrained focus of infection might present with an elevated platelet count, even in the absence of other signs of infection, such as fever or an elevated white blood cell (WBC) count. Furthermore, several observational studies have shown that infection is a common cause of thrombocytosis [1-7]. More generally, these studies have demonstrated that thrombocytosis is usually reactive in nature, i.e., secondary to an underlying inflammatory condition such as infection, malignancy or tissue damage. By contrast, primary thrombocytosis, or an elevation in platelet count due to a myeloproliferative disorder (MPD), is relatively rare.

Although prior studies have established infection as a cause of thrombocytosis, they lack a detailed description of the underlying infections associated with an elevated platelet count. They also offer little information as to laboratory and clinical signs that may point to an infectious etiology.

Thus, in this study, we describe the etiologies of thrombocytosis in 801 adult patients in a general medicine population, with an emphasis on infectious causes. We report the types of infection associated with thrombocytosis and the causative microorganisms and predisposing conditions, as well as the presence of other clinical or laboratory indicators of infection. We also comment on the relationship between treatment of the underlying infection and clinical outcome, including the potential role of thrombocytosis as a clinical marker of unresolved infection. This observational study adds to the understanding of the association of infection and platelet counts, to aid clinicians in the identification and management of patients with thrombocytosis due to infection.

## Materials and Methods

### Clinical setting

The Michael E. DeBakey VA Medical Center (MEDVAMC) is a 520-bed, tertiary care hospital that provides primary medical care for roughly 120,000 veterans and has approximately 15,000 admissions annually. All medical records, including progress notes, nursing notes, consultations, discharge summaries, vital signs, laboratory studies, radiographic and pathologic data, are available electronically.

The protocol was approved by the Institutional Review Board of Baylor College of Medicine and by the Research and Development Committee of the MEDVAMC, and all research activities were conducted at the MEDVAMC / Baylor College of Medicine.

### Identification of patients with thrombocytosis

By querying electronic medical records, we retrospectively identified adult patients (≥ 18 years of age) at the MEDVAMC with documented thrombocytosis (platelet count > 500 × 10^9^/L) between March and December 2005. If a patient had multiple episodes of thrombocytosis in the given period, only the episode with the highest platelet count was included. Patients were excluded if the documented platelet count had no associated clinical notes. All patients who met criteria during the study period were included. Overall, the data from 801 patients were analyzed.

### Sources for data collection

We recorded demographic data, vital signs and laboratory values, as well as predisposing conditions (such as diabetes and chronic liver disease), admission diagnoses/chief complaints and the presence of indwelling prostheses, including vascular catheters. Data for fever, anemia, tachycardia and WBC count were collected from the same day as the platelet count. We also documented available culture data, as well as treatments and patient outcomes.

To determine the etiology of thrombocytosis for each case, we reviewed discharge summaries and clinical notes, including daily progress notes, hematology consultations and infectious diseases consultations. We classified patients as having either primary or secondary thrombocytosis, then subcategorized patients by specific etiology as outlined below; our sources for the following definitions included prior case series of patients with thrombocytosis [2-7].

### Definitions

Patients with primary thrombocytosis had a diagnosis of MPD cited in the medical record, while patients with secondary thrombocytosis had documentation of either an infectious or non-infectious condition known to cause thrombocytosis. Documentation of the etiology of thrombosis was considered adequate if the primary team or consultant arrived at a diagnosis in their notes, or if pathology or cultures confirmed a diagnosis after discharge. Infectious causes included central nervous system, pulmonary, gastrointestinal (GI), genitourinary (GU), skin/soft tissue, osteomyelitis or “other”. Skin colonization with methicillin-resistant *Staphylococcus* aureus was not considered an infection. Non-infectious causes included tissue damage (due to surgery or trauma), malignancy, chronic inflammatory disease, post-splenectomy, drug related, iron deficiency anemia or “other”. Thrombocytosis was considered drug related if the rise in platelets correlated with the use of vinca alkaloids, gemcitabine, iron sulfate, ciprofloxacin, or piperacillin/tazobactam, as these drugs have previously been associated with thrombocytosis [8-10].

In terms of clinical outcomes, patients were classified as having either complete resolution (the underlying cause of thrombocytosis was resolved by the time of discharge), incomplete resolution (the underlying cause of thrombocytosis was still present at the time of discharge, or the patient was readmitted for the same condition within 1 month of discharge), death (the patient expired prior to discharge or within 1 month of discharge from a cause of death clearly related to the cause of thrombocytosis) or unknown (insufficient follow-up available). Patients with an infectious cause of thrombocytosis were classified as being managed with no treatment, with antibiotics alone, with surgery (if the patient underwent a procedure intended to treat the infection), or with a combination of antibiotics and surgery.

### Timing of data collection

Data were collected between April and August 2006. Follow-up was extended for an additional 2.5 years for the 147 patients initially classified as having an indeterminate cause of thrombocytosis, which reduced that number to 59.

### Statistical analysis

Data were entered into a Microsoft Excel database, and all analyses were conducted using SAS statistical software version 9.1 (SAS Institute, Cary, NC). Univariate comparisons of demographic and clinical characteristics of patients with primary and secondary thrombocytosis were conducted. Differences among categorical variables were tested using the chi-square test. Corresponding odds ratios (OR) and 95% confidence intervals (CI) were used to measure the likelihood of an infectious cause of thrombocytosis for various predictor variables. The percentage of patients with each possible etiology of thrombocytosis was calculated (primary, secondary non-infectious, and secondary infectious). Stepwise logistic regression was also performed with whether the etiology of thrombocytosis was infectious or non-infectious (including primary and secondary non-infectious etiologies) as the outcome variable. Covariates included those characteristics defined as significant on univariate analysis and those found to be biologically plausible. Potential predictor variables that were entered in the model were the following (all were yes/no): inpatient status, quadriplegia/paraplegia, indwelling prosthesis, dementia, diabetes, fever, tachycardia, elevated peripheral WBC count, and anemia. Weight loss, hypoalbuminemia and elevated absolute neutrophil count were not included because more than 20% of patients had missing values. Variables were retained in the model if the P value was 0.05 or less. The ability of the model to discriminate those with an infectious versus a non-infectious cause of thrombocytosis was measured by the C statistic, a measure of the area under the receiver operating characteristic curve. Values closer to 1 indicate better discriminatory ability. The goodness of fit of the model was evaluated using the Hosmer-Lemeshow goodness-of-fit statistic. Non-significant values indicate adequate fit of the model to the data.

## Results

### Demographic data

Of 801 patients analyzed, 747 were men (93.3%); and 54 were women (6.7%); the mean age was 62.3 (± 12.4) years. Most patients were White (55.2%) or Black (35.2%), with other racial and ethnic groups represented as follows: Hispanic (6.6%), Asian (0.1%), Native American (0.1%), other (1.0%), and unknown (1.8%). Inpatients made up 58.5% of the study population. As depicted in Table 1, diabetes, malignancy, coronary artery disease, chronic obstructive pulmonary disease (COPD) and alcoholism were all common pre-existing diagnoses, while Human Immunodeficiency Virus (HIV) was rare among subjects.

**Table 1:** Pre-Existing Conditions^a^

Pre-existing condition	Number of patients (n = 801)	Percentage (n = 801)
Diabetes mellitus	243	30.3%
Malignancy	206	25.7%
Coronary artery disease	198	24.7%
COPD	126	15.7%
Alcoholism	121	15.1%
Chronic renal insufficiency	72	9.0%
Chronic liver disease	67	8.4%
Peripheral vascular disease	52	6.5%
Dementia	49	6.1%
Quadriplegia/paraplegia	30	3.7%
HIV	9	1.1%

^a^Each patient in the study could have multiple pre-existing conditions. Abbreviations: COPD = chronic obstructive pulmonary disease; HIV = human immunodeficiency virus.

### Degree of thrombocytosis and time to normalization of platelet count

The platelet count for each subject was recorded as either high (500 - 599 × 10^9^/L), very high (600 - 800 × 10^9^/L) or extreme (> 800 × 10^9^/L). Most patients (53.8%) had a high platelet count, while 33.3% had a very high platelet count. Only 12.9% of subjects had extreme thrombocytosis.

Although most patients’ platelet count normalized within 6 months (63.6%), a substantial portion had unresolved thrombocytosis at the termination of the study (15.1%).

### Etiology of thrombocytosis

Although the elevated platelet count was attributed to multiple causes in some patients, each etiology was considered separately for data analysis, thus bringing the total number of cases to 890 (Table 2). In most cases, thrombocytosis was secondary; primary causes of thrombocytosis were rare. The etiology of thrombocytosis was indeterminate in 7.4% of subjects (after re-review). Essential thrombocythemia (ET) was the most common cause of primary thrombocytosis. Among secondary, non-infectious etiologies, tissue damage was the most common, followed by malignancy and iron-deficiency anemia. The most common infectious causes of thrombocytosis were soft-tissue, pulmonary and GI infections.

**Table 2:** Etiology of Thrombocytosis

Etiology	Number of cases (n = 890)^a^	Percentage of patients with each etiology (n = 801)^a^
Primary	42	5.2%
Essential thrombocythemia	22	
Polycythemia vera	10	
Chronic myelogenous leukemia	1	
Unspecified MPD	9	
Secondary, non-infectious	405	50.6%
Tissue damage^b^	196	
Malignancy	86	
Iron-deficiency anemia	59	
Chronic inflammatory disease^c^	37	
Drug related	26	
Post-splenectomy	12	
Other^d^	23	
Secondary, infectious	384	47.9%
Soft-tissue infection	143	
Pulmonary infection	123	
Gastrointestinal infection	83	
Genitourinary infection	80	
Osteomyelitis	31	
Other^e^	56	
Indeterminate	59	7.4%

^a^Because some patients had multiple etiologies of thrombocytosis, the total number of cases exceeds the number of patients in the study, and percentages add up to greater than 100%; ^b^e.g., trauma, bone fracture, surgery; ^c^e.g., rheumatoid arthritis, systemic lupus erythematosus; ^d^e.g., small-bowel obstruction, bile-duct obstruction, gastric perforation, spinal-cord compression, drug-induced skin rash, knee inflammation, pericarditis and over-correction of the bone marrow; ^e^e.g., bacteremia without a source, intravascular-line infections, endocarditis, wound infections, parotitis, otitis media and externa, odontogenic infection and phlebitis. Abbreviations: MPD = myeloproliferative disorder.

A variety of microorganisms were isolated from patients with infectious causes of thrombocytosis, although culture data were unavailable for some subjects. As shown in Table 3, the most frequently isolated microorganism was methicillin-resistant *Staphylococcus aureus* (MRSA), followed by *Clostridium difficile*. Other common microorganisms included Enterococcus sp., Pseudomonas sp. and *Escherichia coli*.

**Table 3:** Incidence of Isolated Microorganisms

Microorganism	Number of cases
Methicillin-resistant *Staphylococcus aureus*	63
*Clostridium difficile*	48
Enterococcus sp.	37
Pseudomonas sp.	36
*Escherichia coli*	35
Klebsiella sp.	23
Coagulase negative staphylococci (CONS)	22
Methicillin-sensitive *Staphylococcus aureus* (MSSA)	20
Beta-hemolytic streptococci	16
Other streptococci	16
Proteus sp.	11
Bacteriodes sp.	7
Candida sp.	7
Other^a^	26

^a^e.g., Acinetobacter, Actinomyces, Citrobacter, Corynebacterium, Haemophilus influenzae, Mycobacterium, Prevotella, Propionobacterium, Salmonella and Serratia.

### Characteristics of patients with secondary thrombocytosis due to infection

On univariate analysis (Table 4), subjects who were inpatients at the time of thrombocytosis were statistically more likely than outpatients to have an infectious etiology, as were patients with quadriplegia/paraplegia, an indwelling prosthesis, dementia or diabetes, as compared with patients without these characteristics. Vital signs and laboratory values associated with an infectious cause of thrombocytosis included fever, tachycardia, weight loss, hypoalbuminemia, neutrophilia, leukocytosis and anemia.

**Table 4:** Features Significantly Associated With an Infectious Cause of Thrombocytosis by Univariate Analysis

Clinical or laboratory feature	Percentage of patients with an infectious cause of thrombocytosis among patients with or without the specified clinical or laboratory feature		P value for Chi-square analysis
Clinical characteristics	With	Without	
Inpatient status^a^	65.4%	23.0%	< 0.0001
Quadriplegia/paraplegia	80.0%	46.6%	0.0003
Indwelling prosthesis	65.4%	30.1%	< 0.0001
Dementia	73.5%	46.3%	0.0002
Diabetes	56.4%	44.3%	0.0016
Physical exam findings	With	Without	
Fever (temperature ≥ 38 °C)	91.4%	47.5%	< 0.0001
Tachycardia (heart rate > 100 bpm)	65.4%	44.9%	< 0.0001
Weight loss (> 4.55 kg in 3 months)	52.1%	40.6	0.0087
Laboratory features	With	Without	
Albumin < 35 g/L (3.5 g/dL)	57.8%	20.6%	< 0.0001
Absolute neutrophil count > 8 × 10^9^/L	71.7%	37.6%	< 0.0001
White blood cell count > 10 × 10^9^/L	61.6%	33.1%	< 0.0001
Anemia (Hb < 120 g/L) (12 g/dL)	51.0%	42.9%	0.0282

^a^For example, 65.4% of inpatients had an infectious cause of thrombocytosis, whereas 23% of outpatients had an infectious cause of thrombocytosis. Abbreviations: C = Celsius; bpm = beats per minute; Hb = hemoglobin.

On multivariate analysis (using a stepwise logistic-regression model), the following characteristics were predictive of an infectious cause of thrombocytosis: fever, inpatient status, dementia, leukocytosis, diabetes and tachycardia (Table 5). Of note, weight loss, hypoalbuminemia, and elevated absolute neutrophil count were not included in the stepwise logistic-regression model due to missing values for more than 20% of patients; otherwise, all characteristics identified as significant on univariate analyses (Table 4) were entered into the model.

**Table 5:** Multivariate Analysis^a^: Features Which Increased the Likelihood of Having an Infectious Cause of Thrombocytosis Versus a Non-Infectious Cause

Clinical or laboratory feature	Odds ratio	95% Wald Confidence Limit
Fever (temperature ≥ 38 °C)	6.64	1.86 - 23.73
Inpatient status	5.04	3.51 - 7.25
Dementia	2.44	1.17 - 5.06
White blood cell count > 10 × 10^9^/L	2.13	1.52 - 2.99
Diabetes	1.67	1.16 - 2.39
Tachycardia (heart rate > 100 bpm)	1.66	1.08 - 2.54

^a^A stepwise logistic regression model was used for multivariate analysis; see text for details. C statistic = 0.783. Hosmer and Lemeshow goodness-of-fit statistic, P = 0.3149. Abbreviations: C = Celsius; bpm = beats per minute.

### Etiology and degree of thrombocytosis

Patients with primary thrombocytosis had a higher mean platelet count (857.3 × 10^9^/L) than patients with secondary, non-infectious (654.1 × 10^9^/L) or infectious causes (644.2 × 10^9^/L). In fact, patients with platelet counts > 800 × 10^9^/L (extreme thrombocytosis) were more likely to have an MPD (OR, 4.75; 95% CI 2.45 - 9.20; P < 0.0001). Apart from this finding, the degree of thrombocytosis did not correlate with the etiology of thrombocytosis or with a particular microorganism.

### Etiology and time to normalization of platelet count

Patients with primary thrombocytosis were more likely to have a prolonged elevation in platelet count (time to normalization > 1 month) (OR, 8.86; 95% CI, 3.11 - 25.21; P < 0.0001). Patients with secondary, non-infectious etiologies were also more likely to have a prolonged time to normalization (OR, 1.53; 95% CI, 1.13 - 2.07; P = 0.0054). By contrast, patients with an infectious etiology were less likely to have prolonged thrombocytosis (OR, 0.35, 95% CI, 0.26 - 0.48; P < 0.0001).

### Clinical outcome

Most patients experienced either complete or partial resolution of the underlying cause of thrombocytosis (39.2% and 40.7%, respectively), but a minority of patients (7.9%) expired. In 12.2% of cases, the outcome was unknown, based on the medical record. Of patients with an infectious cause of thrombocytosis, 13.3% expired, compared with 4.4% of patients with non-infectious causes of thrombocytosis; in other words, patients with an infectious cause of thrombocytosis were more likely to die than patients with a non-infectious cause of thrombocytosis (OR, 3.34; 95% CI, 1.83 - 6.09; P < 0.0001).

Because we had anecdotally observed that patients with an undrained focus of infection had prolonged clinical courses and persistent thrombocytosis, we investigated the relationship between treatment modality and clinical outcome. Of patients with an infectious cause of thrombocytosis, 62.7% of those treated with surgery had complete resolution at discharge, compared with 46.3% of patients who received antibiotics alone or no treatment; in other words, patients with surgical management of an infection were more likely to have complete resolution of the underlying infection (OR, 1.94; CI, 1.23 - 3.09; P = 0.0043).

## Discussion

In this study, we reviewed records of 801 patients with an elevated platelet count, with the goal of identifying clinical characteristics that point to an infectious cause of thrombocytosis. Our results corroborate prior findings [1-7] but also provide novel insights into the microbiology and clinical outcomes associated with infectious causes of thrombocytosis.

Secondary thrombocytosis was far more common than primary thrombocytosis, and the frequency of infectious and non-infectious causes was roughly equivalent. These findings are supported by prior investigations of adult patients with thrombocytosis [1-7].

Essential thrombocythemia was the most common MPD in this study, in keeping with at least 1 prior series [5]. Also consistent with prior studies, patients with a MPD had a higher mean platelet count, and patients with extreme thrombocytosis (> 800 × 10^9^/L) were more likely to have a MPD [1, 3, 7, 11]. Although the mechanism of thrombocytosis in MPD is still under investigation, purported mechanisms involve genetic mutations that result in hypersensitivity to proteins that stimulate platelet production [12-17]. These genetic alterations may explain the more dramatic and prolonged elevation in platelet count associated with MPD.

Among secondary, non-infectious causes of thrombocytosis, tissue damage was the most common etiology, followed by malignancy and iron-deficiency anemia, again in keeping with prior series [2, 4, 6]. Patients with secondary, non-infectious etiologies were more likely to have prolonged thrombocytosis (> 1 month). This result likely reflects the chronic nature of many non-infectious etiologies (for example, malignancy, iron-deficiency anemia, chronic inflammatory disease).

Skin/soft-tissue and pulmonary infections were the most common infectious causes of thrombocytosis in this study, though GI and GU infections were also frequent. Similarly, a recent survey of thrombocytosis in an adult Turkish population showed that urinary tract and respiratory infections were the leading diagnoses, followed by “connective tissue” and GI infections [1]. A series by Griesshammer et al in 1999 showed pneumonia to be the most frequent infectious cause of thrombocytosis, while soft-tissue and GI infections were the next most common diagnoses [5]. A smaller study by Robbins and Barnard in 1983 showed pulmonary infections to be most common, followed by “surgical” infections [6].

To our knowledge, this is the first study of adult patients with thrombocytosis to provide information on etiologic microorganisms. MRSA and *C. difficile* were most commonly isolated in this study, which may be due to either a high prevalence of MRSA and *C. difficile* infections among the study population, or the relative ease with which MRSA and *C. difficile* can be identified with standard microbiological techniques. On the other hand, these organisms may have an increased propensity to stimulate reactive thrombocytosis. Interestingly, in a recent study of 162 patients with *C. difficile*, 22% were found to have an elevated platelet count, and toxin A was isolated more frequently in patients with both leukocytosis and thrombocytosis, suggesting that the pretest probability for *C. difficile* infection may be improved by thrombocytosis [18]. Convincingly, in vitro data suggest that the quantity of platelets is important for controlling *S. aureus* infection [19] and observational data in humans has shown that thrombocytopenia is associated with poorer outcome in *S. aureus* bacteremia [20].

Additional research would be required to investigate the tendency and/or mechanisms for MRSA and *C. difficile* to cause an elevated platelet count.

As in prior series [5-7], patients with infectious etiologies had a faster normalization of the platelet count, highlighting the acuity of infectious causes of thrombocytosis. Notably, patients with an infectious etiology were also more likely to die in this study compared with patients with non-infectious causes of thrombocytosis. In fact, the mortality rate among patients with infectious etiologies was strikingly high (13.3%). Among patients with an infectious cause of thrombocytosis, those treated with surgery were more likely to have complete resolution at the time of discharge. Although the retrospective study design limits the interpretation of this finding, the data support our observations of patients with prolonged clinical courses and persistent thrombocytosis related to an undrained focus of infection.

The main limitations in this study are those associated with a retrospective investigation: the available data varied between patients, and we could not assess causative relationships. In addition, while the Michael E. DeBakey VA Medical Center serves a large population with a wide array of illnesses, the demographic homogeneity and primarily male population may have influenced our results.

Overall, our findings suggest that an infectious cause of thrombocytosis should be considered in patients at risk for infection (namely, inpatients or those with quadriplegia/paraplegia, an indwelling prosthesis, dementia or diabetes) or in patients whose presenting complaints are suggestive of common infections (namely, skin, pulmonary, GI, GU). Clinical or laboratory evidence of severe illness (fever, tachycardia, weight loss, hypoalbuminemia, neutrophilia, leukocytosis or anemia) should also raise the index of suspicion for an infectious cause. Although most infections result in transient thrombocytosis, a persistently elevated platelet count should prompt a work up for an inadequately treated infection. Empiric treatment for soft-tissue and GI infections in patients with thrombocytosis may warrant coverage for MRSA and *C. difficile*, since these were the most commonly isolated microorganisms in this study. Appropriate surgical management of infection may be associated with a better outcome. Finally, since infectious causes of thrombocytosis were correlated with an increased risk of death, it seems prudent to recommend deciphering the cause of thrombocytosis and treating if possible.
